# Performance upgrade in the JAEA actinide science beamline BL23SU at SPring-8 with a new twin-helical undulator

**DOI:** 10.1107/S0909049512006772

**Published:** 2012-03-17

**Authors:** Yuji Saitoh, Yoshihiro Fukuda, Yukiharu Takeda, Hiroshi Yamagami, Sunao Takahashi, Yoshihiro Asano, Toru Hara, Katsutoshi Shirasawa, Masao Takeuchi, Takashi Tanaka, Hideo Kitamura

**Affiliations:** a Quantum Beam Science Directorate, Japan Atomic Energy Agency, 1-1-1 Kouto, Sayo, Hyogo 679-5148, Japan; b Department of Physics, Kyoto Sangyo University, Kyoto 603-8555, Japan; c Japan Synchrotron Radiation Research Institute, 1-1-1 Kouto, Sayo, Hyogo 679-5198, Japan; d RIKEN Harima Institute, 1-1-1 Kouto, Sayo, Hyogo 679-5148, Japan

**Keywords:** twin-helical undulator, helicity switching, soft X-ray beamline, X-ray magnetic circular dichroism, uranium compounds

## Abstract

The soft X-ray beamline BL23SU at SPring-8 has undergone an upgrade with a twin-helical undulator of in-vacuum type to enhance the experimental capabilities of the endstations. The new light source with a fast helicity-switching operation allows not only the data throughput but also the sensitivity in X-ray magnetic circular dichroism (XMCD) to be improved. The operational performance and potential are described by presenting XMCD results of paramagnetic β-US_2_ measured with a 10 T superconducting magnet.

## Abstract 2

The commissioning studies and an application to a uranium compound with the helicity-switching mode of a new twin-helical undulator installed in soft X-ray beamline BL23SU at SPring-8 are described.

## 1. Introduction

The JAEA actinide science beamline, BL23SU, built and operated by Japan Atomic Energy Agency (JAEA), is a soft X-ray beamline at the SPring-8 synchrotron facility with its first light being received in 1998 (Saitoh *et al.*, 2001 ▶). The primary aim of BL23SU is to investigate the electronic structures of uranium-based compounds and related materials using soft X-ray photoemission (SX-PES) and magnetic circular dichroism (XMCD) spectroscopies with a focus on superconductivity and magnetism. The uranium compounds display a variety of intriguing phenomena, which is considered to be due to complex many-body interactions between the valence-electron states. Recently, superconductivity has been discovered in heavy-fermion ferromagnets, such as UGe_2_, URhGe, UCoGe and UIr [for a recent review see, for example, Pfleiderer (2009 ▶)], and therefore XMCD as well as SX-PES studies are required for a comprehensive understanding of the nature of the 5*f* electrons. With the availability of synchrotron radiation sources, XMCD has been developing as a probe of local (element- and angular-momentum-specific) spin and orbital magnetic moments (Funk *et al.*, 2005 ▶).

**Figure 1:**
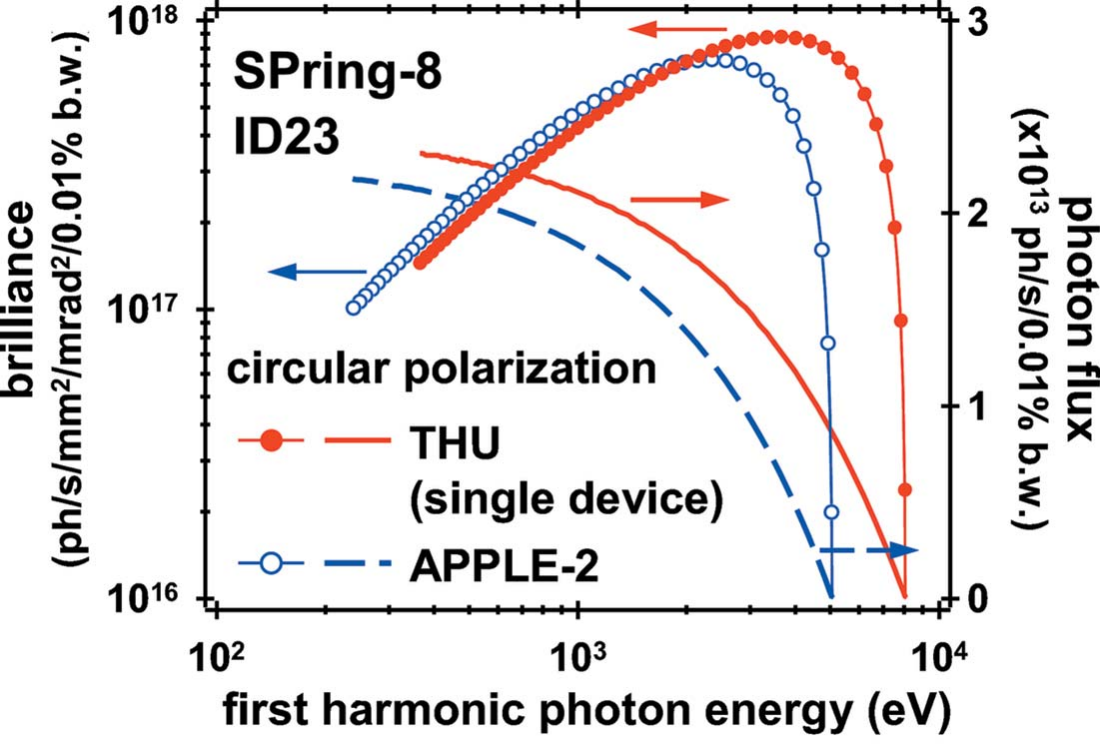
Source brilliance and photon flux in circular polarization mode calculated for the single device of the twin-helical undulator (THU) and the replaced APPLE-2 device of ID23 for SPring-8 100 mA operation.

The BL23SU beamline was initially commissioned with an APPLE-2-type undulator with flexible polarization properties (Sasaki, 1994 ▶), in which the four rows of periodic magnets are arranged with two rows above and two rows below the stored electron orbit plane. The length (*L*) of the undulator was 2 m and located at the upstream half of a 5.7 m straight section. The periodic length (λ_u_) of the undulator was 12 cm and the number of periods (*N*_p_) was 16. It covered the energy range down to 240 eV in circular polarization mode with a minimum gap of 25 mm (see Fig. 1 ▶). In this device, photon helicity was switched by the longitudinal mechanical shift of the magnetic arrays (*i.e.* changing the magnetic row phase), and the helicity switching at each photon energy of an XMCD spectrum was an important technical approach for high-accuracy XMCD experiments (Agui *et al.*, 2001 ▶). A local feed-forward correction system for the orbit distortion at the switching time up to around 2.5 s between opposite helical polarizations (the phase parameter in its pure helical mode was roughly proportional to the gap) has been developed (Nakatani *et al.*, 2005 ▶). This system allowed us to perform efficient XMCD measurements using a superconducting magnet under fixed sample conditions (Okamoto *et al.*, 2004 ▶). This superconducting magnet requires a long-field reversing time and therefore point-by-point field reversal was impractical. The frequent mechanical motion of the magnet arrays, however, resulted in a high percentage of beamline downtime caused by component failures, thus creating some problems with maintenance and parts replacement. With regard to the SX-PES experiments, a clear advantage of the circular polarization mode over the linear polarization mode was the higher stability in photon energy and intensity, reflecting the much lower heat load on optical components (Kincaid, 1977 ▶).

Meanwhile, the twin-helical undulator of BL25SU (Hara *et al.*, 1998 ▶), ID25, pioneered at SPring-8, has provided reliable and productive operations for helicity-switching dichroism experiments (Muro *et al.*, 2005 ▶; Nakamura *et al.*, 2005 ▶; Hayashi *et al.*, 2011 ▶) with almost complete circular polarization (*P*_c_) and full polarization reversal (Hirono *et al.*, 2005 ▶) for a switching frequency up to 10 Hz (Shirasawa *et al.*, 2004 ▶). The twin-helical undulator consists of two in-line helical undulators and five kicker magnets. In these helical undulators the six rows of periodic magnets are arranged with three rows above and three rows below the electron orbit plane. Each of the helical undulators is an out-of-vacuum device (λ_u_ = 12 cm, *N*_p_ = 12) with a minimum gap of 20 mm to cover the energy range down to 120 eV. In helicity-switching mode, the kicker magnets alternately separate the photon beams horizontally by 0.3 mrad in order to minimize the overlap between the cones of fundamental radiation with opposite helicities from the two devices. Coordinated scanning of the undulator gaps and the monochromator has also been successfully demonstrated in this mode. The angular aperture for the central radiation cones along the beamline axis is defined by front-end *XY* slits (Oura *et al.*, 1998 ▶). A detailed description of the helicity switching in the twin-helical undulator has been given previously (Hara *et al.*, 2003 ▶).

This type of undulator was adopted for BL23SU using in-vacuum helical undulators, each with *N*_p_ = 17, of λ_u_ = 7.52 cm in order to enhance the photon flux above the N 1*s* absorption edge. Fig. 1 ▶ shows a comparison of the brilliance and photon flux in circular polarization mode between the replaced APPLE-2 undulator (*L* = 2 m) and the single new undulator (*L* = 1.3 m) calculated using the program *SPECTRA* (Tanaka & Kitamura, 2001 ▶). Unlike ID25, the central row of the magnetic array, namely the phase of the magnetic field, is fixed in ID23 because of the difficulty of having linear guide systems in an UHV environment. Thus the polarization of the soft X-ray radiation was restricted to be circular. The two undulator gaps of the new ID23 can be independently adjusted down to 8 mm at present, covering photon energies down to 370 eV. Small gaps of the in-vacuum undulator allow us to reduce λ_u_, thereby increasing *N*_p_, being proportional to the photon flux output.

The maximum frequency of helicity switching is 10 Hz, which is limited by the diagnostics of the electron beam orbit. To maintain coexistence with other beamlines, the variation of the electron beam orbit owing to helicity switching is required to be less than ±1 µm r.m.s. Since the operation of the kicker magnets is independent of the undulator gaps, a simple feed-forward correction scheme is employed for the orbit correction. The twin-helical undulator of ID23 can also be applied to XMCD experiments at much higher switching frequency by using a fast mechanical chopper and angularly separated dual photon beams, in which the kicker magnets generate a stationary orbit bump (Sawhney *et al.*, 1997 ▶).

The use of an electromagnetic undulator (Freeland *et al.*, 2002 ▶) has an important advantage over the twin-helical undulator for an identical source point of both helicities. However, a fast orbit correction feed-back, which is currently not adopted in the SPring-8 storage ring, is necessary, otherwise feed-forward tables should be made for each electromagnet current and the orbit correction becomes too complicated and less accurate (Oura *et al.*, 2007 ▶). Therefore it was not considered as a new ID23.

Soft X-ray magnetic linear dichroism (XMLD) spectroscopy measurements were not included in the scientific case of BL23SU because both the experiment and interpretation were considered to be more challenging than for XMCD. For this reason, no XMLD experiments had been conducted with the APPLE-2 device.

Recently, the helicity switching capability at a frequency of 1 Hz has reached operational status as a first step. In this operation each of the two polarized beams is alternately supplied to the beamline for a period of 0.3 s with a transient time of 0.2 s. We report here its performance for XMCD experiments as well as some recent upgrades of BL23SU.

## 2. Beamline description

**Figure 2:**
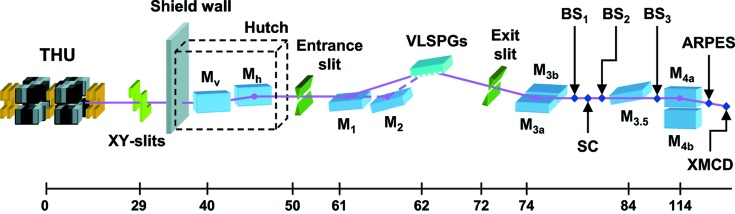
Schematic of the BL23SU beamline at SPring-8. The scale indicates the distance in meters from the center of the twin-helical undulator (THU). M_v_: vertical condensing mirror; M_h_: horizontal collimating mirror; M_1_ and M_2_: vertical focusing mirrors; M_3a_: vertical collimating mirror; M_3b_, M_3.5_, M_4a_ and M_4b_: refocusing toroidal mirrors; BS_1_–BS_3_: biophysical spectroscopy stations; SC: surface chemistry station. The M_3b_ and M_3.5_ mirrors are used for experiments at the SC and BS_3_ stations, respectively.

A schematic overview of the main beamline components of BL23SU is shown in Fig. 2 ▶. The undulator radiation is transported by the front-end within the storage-ring shield wall to the downstream optics. In order to stop the off-axis beam deflected by the kicker magnets, some of the front-end components upstream of the *XY* slits, including heat-absorbing photon masks, have been replaced by ones with wider acceptances.

The optical system of BL23SU, as detailed elsewhere (Saitoh *et al.*, 2001 ▶), consists of prefocusing mirrors (M_v_ and M_h_) and a varied-line-spacing plane-grating monochromator (VLSPGM, from S_1_ to S_2_) equipped with an entrance slit and refocusing mirrors (M_3a,b_, M_3.5_, M_4a,b_). This undulator replacement did not need the modification of a hutch enclosure housing the prefocusing mirrors. The VLSPGM was designed to provide an operational range from 0.2 to at least 1.5 keV with mechanically ruled blazed gratings and achieved a resolving power in excess of 1 × 10^4^ at the nitrogen *K*-edge in full aperture operation for the circular polarization mode of the APPLE-2 undulator (Saitoh *et al.*, 2001 ▶). However, the photon flux available turned out to be insufficient for soft X-ray angle-resolved photoemission spectroscopy (SX-ARPES) successfully demonstrated at the BL25SU beamline (Suga *et al.*, 2004 ▶). Recent SX-ARPES measurements at BL23SU with energy resolutions down to 0.1 eV (Fujimori *et al.*, 2007 ▶) have been facilitated by the substitution of a holographic ruled grating with a central groove density of 600 lines mm^−1^ into the VLSPGM, which resulted in practically four-fold improvement in throughput while providing a resolving power better than 1 × 10^4^ up to 1 keV.

**Figure 3:**
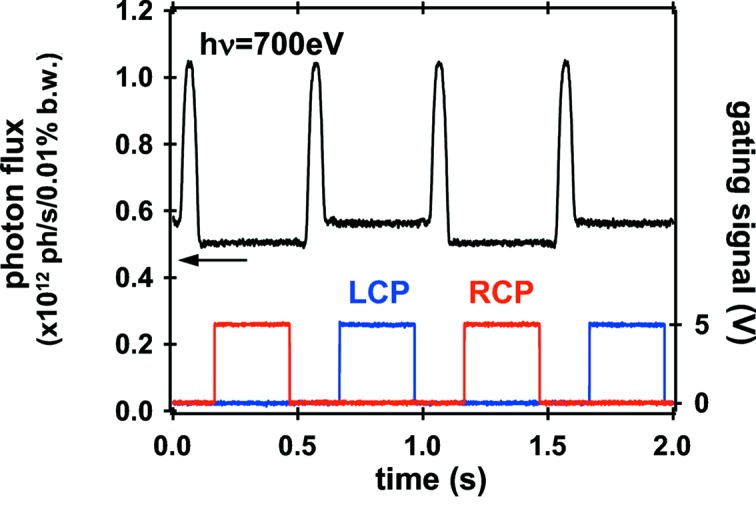
Photon flux during the helicity-switching mode measured for *h*ν = 700 eV at the XMCD station together with the gating TTL signals for the experiments.

After the installation of the new twin-helical undulator, the optical components were slightly adjusted to accommodate a change in the source centers by 1 m by maximizing the transmitted flux at the highest resolution of the VLSPGM using both undulator beams. In this case, monochromatic flux was almost twice that of the APPLE-2 device, as expected, achieving ∼1 × 10^12^ photons s^−1^ (0.01% bandwidth)^−1^ at 700 eV (see Fig. 3 ▶). The enhanced flux is quite useful, not only for SX-ARPES measurements (Kawasaki *et al.*, 2011 ▶) but also for surface reaction analyses at the SC station (Teraoka & Yoshigoe, 2001 ▶) and biological spectroscopies at the BS_1_–BS_3_ stations (Fujii *et al.*, 2009 ▶; Yokoya & Akamatsu, 2001 ▶; Ukai *et al.*, 2009 ▶).

More recently, the SX-ARPES analyzer of a Scienta SES 2002 has been calibrated by modifying the voltage tables in a collaborative research with the Osaka University group of Sekiyama, Kiss *et al.* This calibration provided its original specification in angular resolution and effective angular window, which is accompanied by an enhancement in transmission and detection efficiency by a factor of at least six, thereby reducing data collection times and increasing throughput (Sekiyama *et al.*, 2012 ▶).

The XMCD endstation terminates BL23SU at approximately 120 m from the light source. The experimental UHV chamber is equipped with the superconducting magnet and a sample holder attached to a liquid-He cryostat reaching a sample temperature of 4.5 K. This magnet provides a variable field parallel to the photon beam direction in the range of ±10 T with a sweeping rate up to 1 T min^−1^. XMCD spectra are obtained in total electron yield mode by measuring the sample drain current (*I*) normalized by incident photon flux (*I*_0_) monitored with a Au-coated SiO_2_ refocusing toroidal mirror, M_4a_, or a Au-coated mesh between the M_4a_ mirror and the XMCD station. Each of the signals *I*_0_ and *I* is converted by a current amplifier into a voltage which is subsequently converted to a frequency. These signals are fed into separate counters for individual polarization. In the helicity-switching mode the signals are integrated during time periods determined by gating TTL (transistor–transistor logic) signals from the undulator control system. In addition to this, without the reconnection of signal cables, on-the-fly scanning mode (constant velocity motion of the VLSPG and either undulator gap) with a typical rate of 40 eV min^−1^ has been implemented (Takeda *et al.*, 2008 ▶). This scanning mode was not effective for the APPLE-2 undulator, because the gap scan had been limited to a phase parameter of 0 mm, corresponding to the horizontal linear polarization mode.

## 3. Experimental observations

Fig. 3 ▶ shows the photon flux measured at the XMCD endstation during the 1 Hz-switching mode for a photon energy (*h*ν) of 700 eV along with the received gating TTL signals for left- and right-handed circularly polarized (LCP and RCP) light. The beam-defining aperture, located at approximately 29 m from the center of the light source, was unchanged from 2.5 mm × 2 mm (horizontally × vertically) that had been determined for SX-ARPES experiments. Both of the helical undulators were set to the same gap to maximize the photon flux. During the switching transients, both of the beams with opposite helicities pass through the beamline. The change in intensity of approximately 10% for each helicity, which was measured to be fairly independent of the first-harmonic photon energies, is ascribed to the difference in angular acceptance of the beam-defining aperture owing to the positional difference of 2 m between the two undulator centers. This is because the calculated flux transmitted through the aperture using the *SPECTRA* program adequately accounts for these behaviors.

**Figure 4:**
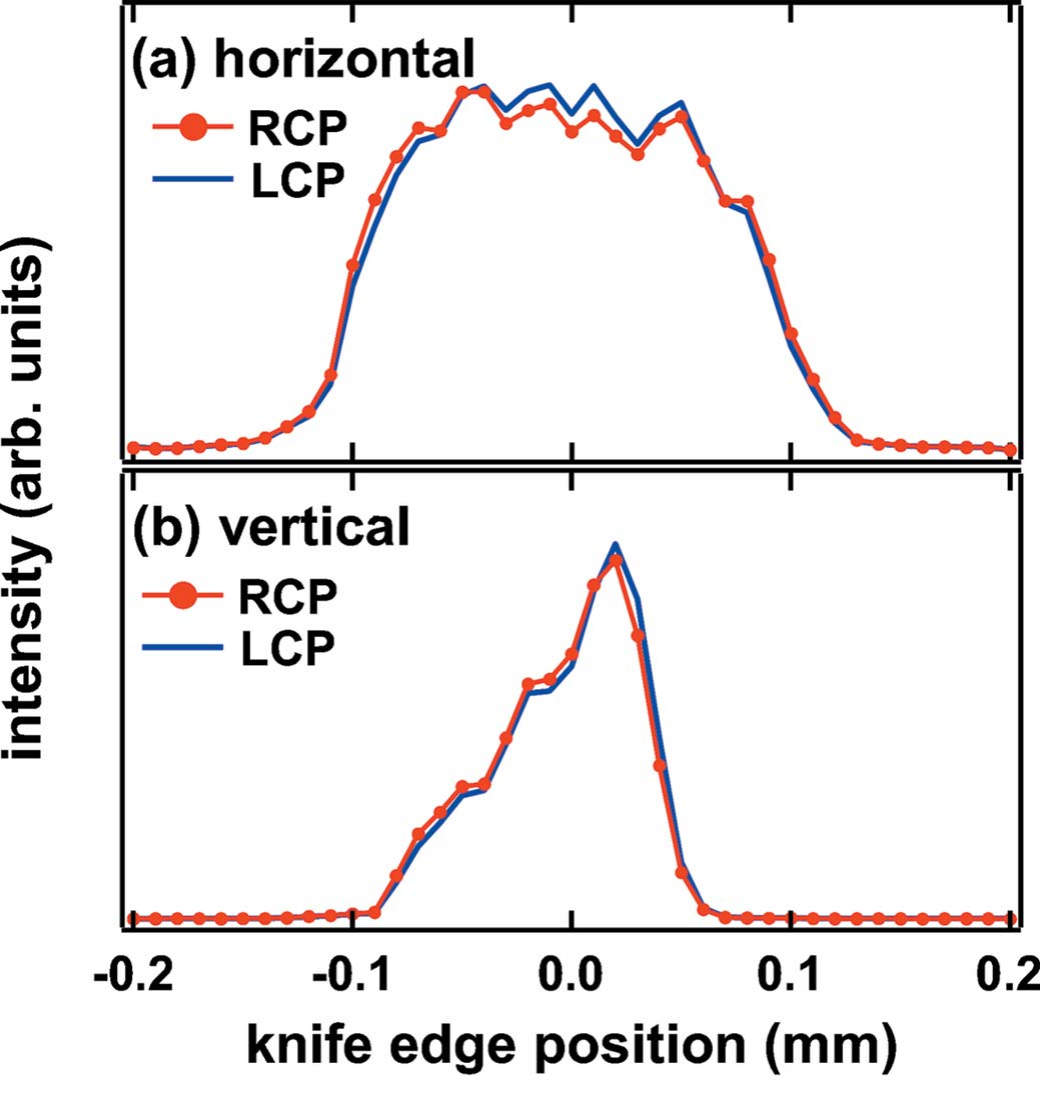
Horizontal (*a*) and vertical (*b*) beam spot profiles in the helicity-switching mode at *h*ν = 700 eV measured at the XMCD station.

Fig. 4 ▶ shows the intensity profiles of the focused beam spots at the XMCD station obtained at *h*ν = 700 eV while varying either vertical or horizontal knife-edge position. The focus spots are produced by the refocusing toroidal M_4a_ mirror which imaged the light source (exit slit width) in the horizontal (vertical) direction with a nominal demagnification of about 6 (1/3). The width of the VLSPGM exit slit was set to 20 µm. These measurements were conducted with the same beam intensity by detuning the downstream LCP undulator gap slightly. The LCP and RCP beams are focused on almost a common spot without additional optical alignment. The subtle difference has virtually no influence on dichroic experiments as shown below.

**Figure 5:**
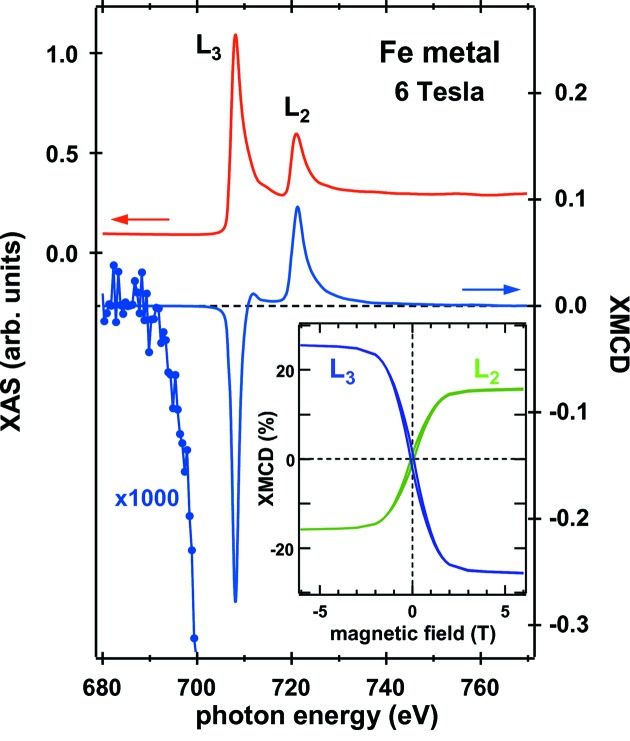
XAS and XMCD spectra at the *L*_2,3_ edges of Fe metal at room temperature at 6 T. The inset shows the XMCD hysteresis loops for the *L*_2,3_ peak energies.

The XMCD performance was tested on a polycrystalline Fe (purity 99.99%) sample at room temperature. Fig. 5 ▶ shows the photon-flux-normalized Fe *L*_2,3_ XAS (μ_L_ + μ_R_) and XCD (μ_L_ − μ_R_) spectra measured with an energy resolution of 70 meV for a fixed magnetic field of 6 T, in which both helicities in one cycle were used for every energy point. Here, μ_L_ (μ_R_) is the X-ray absorption for LCP (RCP) light. The *L*_3_ XAS intensity from the pre-edge background is normalized to unity for ease of comparison with the results obtained by Chen *et al.* (1995 ▶) as described below. The noise level of the measured XMCD spectrum is approximately ±0.03% of the XAS intensity, corresponding to an order of magnitude improvement in sensitivity in our beamline. This sensitivity permits the XMCD onset to be observed at about 18 eV below the *L*_3_ edge.

A closer look at the XMCD data by use of the APPLE-2 device shows that the XMCD sensitivity was generally limited by uneven signals taken immediately following the helicity switching performed at every two measurement points (Agui *et al.*, 2001 ▶). In conventional XAS measurements without helicity switching, the noise level was suppressed to ±0.02% or less of the averaged XAS intensity. The signal variations in the previous XMCD spectra were most likely due to a significant increase in heat load on the optical components caused by the linear polarization mode during the helicity switching.

The inset in Fig. 5 ▶ shows the XMCD asymmetry [(μ_L_ − μ_R_)/(μ_L_ + μ_R_)] recorded at the *L*_3_ (708.1 eV) and *L*_2_ (721.3 eV) edges while sweeping the applied magnetic field. No correction has been made to the curves. The XMCD asymmetry is known to be proportional to the element-specific magnetization projected along the incident photon wavevector (Chen *et al.*, 1993 ▶). In addition, switching the field direction is equivalent to reversing the helicity of the incoming beam for ferromagnetic Fe. The magnetization curves indicate the same circular polarization rate (|*P*_c_|) of the two beams as well as the full saturation of the Fe moment at 6 T, because the XMCD signals for each helicity on reversing magnetization are the same.

The measured XMCD effect is consistent with the previous results of transmission experiments for magnetically saturated Fe films with a correction for the incomplete *P*_c_ (Chen *et al.*, 1995 ▶). In addition, the XMCD asymmetry at 1.4 T is precisely equal to that obtained at BL25SU (Muro *et al.*, 2005 ▶). The spectroscopy results clearly show that the helicity-switching operation of the twin-helical undulator is well suited for high-precision XMCD studies.

**Figure 6:**
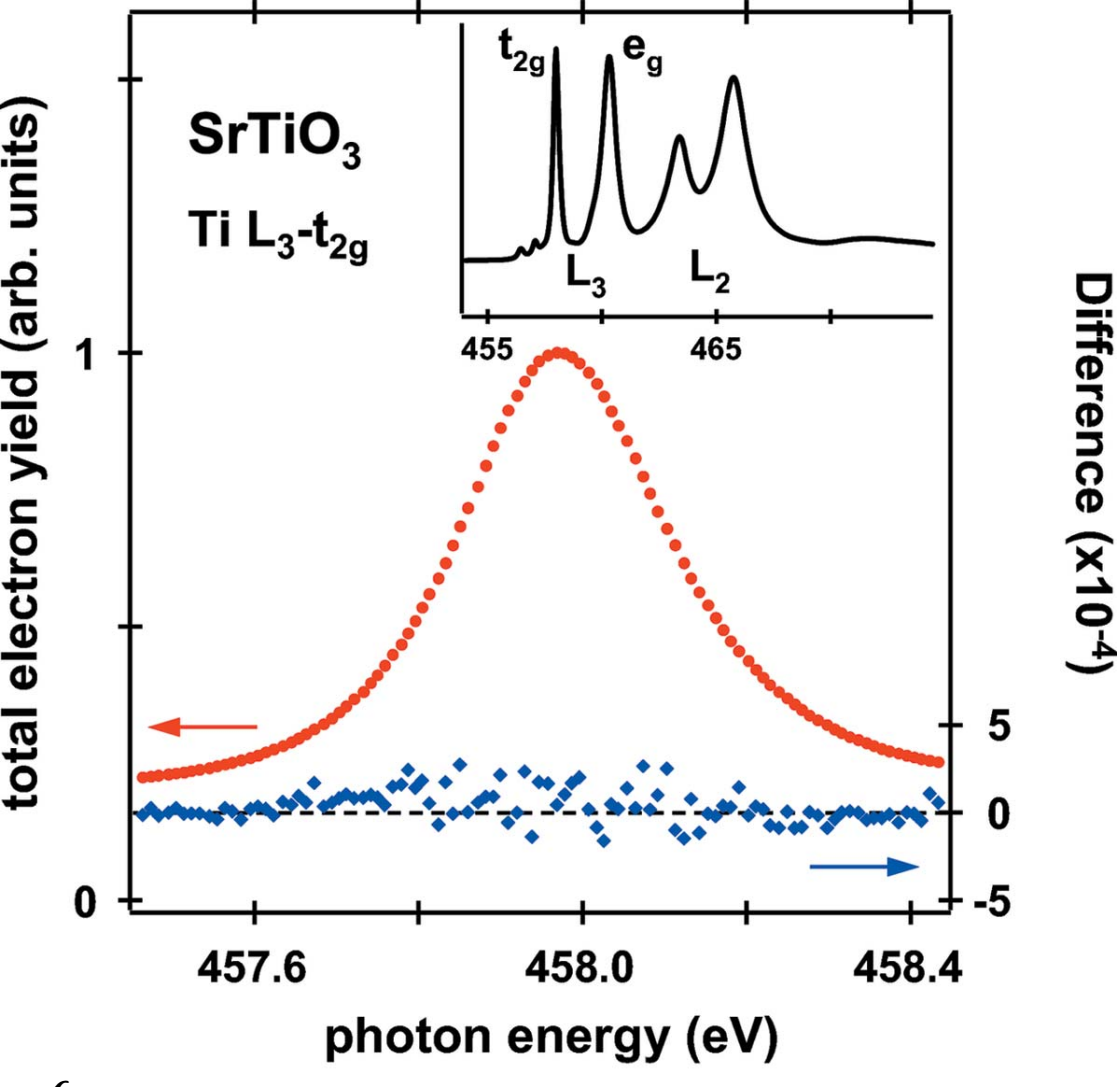
Absorption measurements for the Ti *L*_3_–*t*_2g_ transition of SrTiO_3_ at room temperature. Sum (μ_L_ + μ_R_; circles) and difference (μ_L_ − μ_R_; diamonds) of the two intensities with opposite helicities. The inset shows the overview spectrum for the Ti *L*_2,3_ edges.

When the effective source point for LCP and RCP light is slightly different, this can transform into an energy difference even for a given monochromator setting. The energy shift was examined by the measurement of the Ti 2*p*_3/2_–3*d* (*L*_3_–*t*_2*g*_) absorption spectra of a non-magnetic SrTiO_3_ sample as shown in Fig. 6 ▶. These spectra were taken at room temperature without applying a magnetic field for an energy resolution of 40 meV, in which both helicities in one cycle were used at every energy point. In the difference spectrum, no evidence of dichroic behavior is observed within the detection limit. An energy difference between the two beams will yield a derivative-shaped contribution to the difference spectrum. The energy difference is estimated to be not more than 2 meV from the Voigt fit to the μ_L,R_ spectra.

## 4. Application to a uranium compound

**Figure 7:**
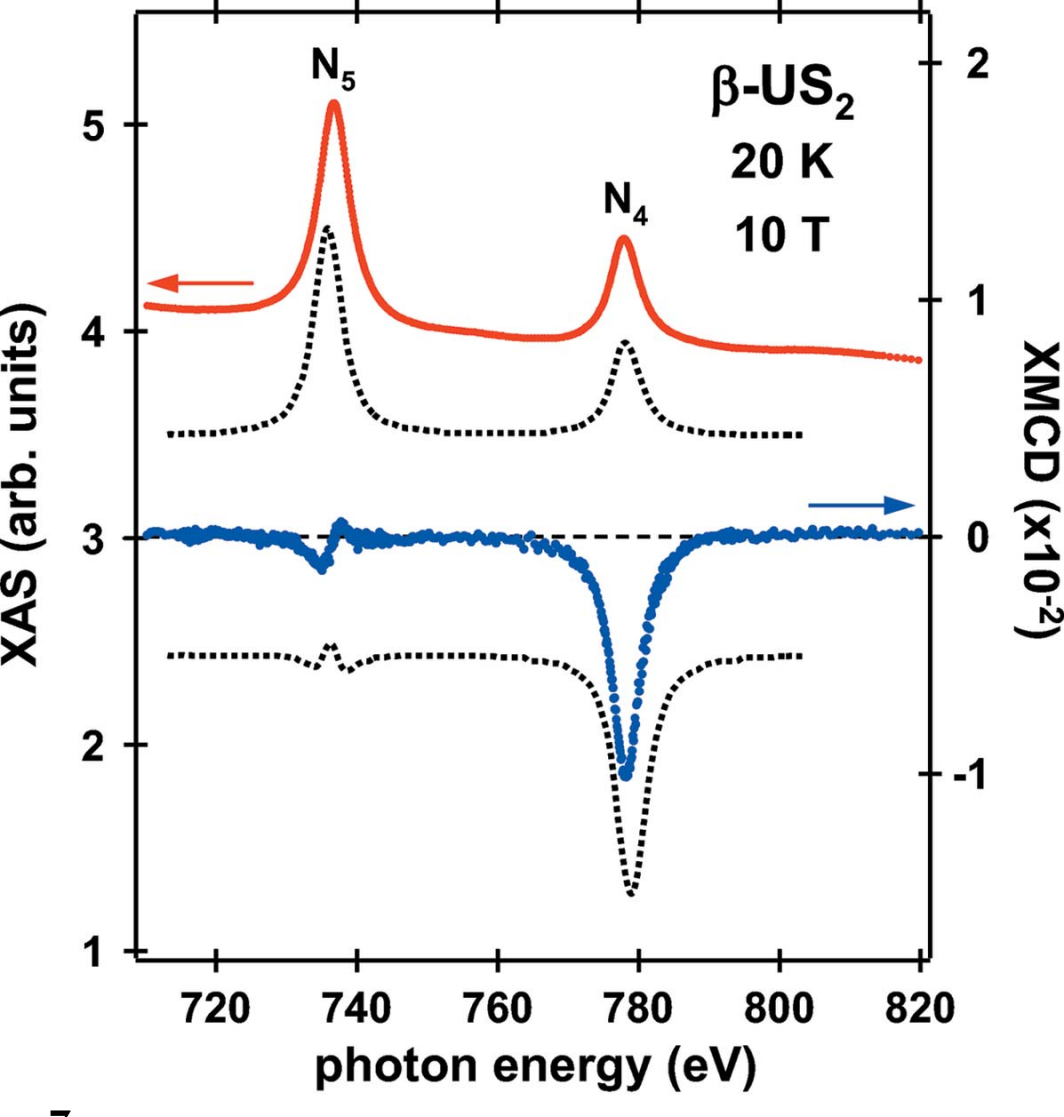
Uranium *N*_4,5_ XAS and XMCD spectra obtained from paramagnetic β-US_2_ at 20 K for an external field of 10 T in comparison with atomic calculations for a 5*f* ^2^ ground-state configuration (dotted curves). The theoretical curves have been offset vertically for clarity.

As an example of investigations that were previously un­approachable, the XAS and XMCD spectra at the U *N*_4,5_ edges in a paramagnetic β-US_2_ single crystal measured along the 〈010〉 direction at 20 K in an applied field of 10 T are shown in Fig. 7 ▶ (sample provided by Advanced Science Research Center, JAEA). These spectra prove U 5*f* states through the dipole allowed 4*d*_5/2_ → 5*f*_5/2,7/2_ (*N*_5_) and 4*d*_3/2_ → 5*f*_5/2_ (*N*_4_) transitions. β-US_2_ is a semiconductor and the U^4+^ (5*f* ^2^) ionic scheme provides a good qualitative description of its magnetic behavior (Ikeda *et al.*, 2009 ▶). So far, no detailed XMCD investigations of U compounds with formally 5*f* ^2^ ground state have been reported. The uranium magnetic moment projected along the photon wavevector is of the order of 0.1 μ_B_ under the experimental conditions, resulting in an XMCD variation of only 0.15% of the XAS intensity at the *N*_5_ edge. In order to eliminate any experimental artifacts arising from system errors, the XMCD spectrum was measured for opposite orientations of the applied magnetic field and the resulting spectra were averaged. Each of the spectra was acquired in two cycles of the helicity switching at every energy point with an energy resolution of ∼125 meV.

Application of the well known sum rules to the XMCD spectrum (Thole *et al.*, 1992 ▶; Carra *et al.*, 1993 ▶) provides valuable information on the 5*f* magnetic moments. The sum rules relate the integrals of the XMCD signal over the *N*_5_ edge (*p*) and over both *N*_5_ and *N*_4_ edges (*q*) to ground-state magnetic properties as
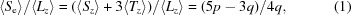
where 〈*L_z_*〉, 〈*S_z_*〉 and 〈*T_z_*〉 are the expectation values of the orbital angular momentum, spin angular momentum and the magnetic dipole term in the magnetization direction, respectively. 〈*S*_e_〉 is referred to as the effective spin. The ratio 〈*S*_e_〉/〈*L_z_*〉 is instructive for the analysis of data on magnetically unsaturated samples and is independent of *P*_c_. From the measured XMCD spectrum, we derive 〈*S*_e_〉/〈*L_z_*〉 = −0.63 ± 0.02, which is in agreement with an atomic 5*f* ^2^ value of −0.66 calculated in intermediate coupling mechanisms for the angular momenta much better than that for *LS* coupling of −0.46 (Collins *et al.*, 1995 ▶; van der Laan & Thole, 1996 ▶). Here we ignored in this analysis the so-called saturation effects in the total electron yield detection (Nakajima *et al.*, 1999 ▶), which have not been investigated for U *N*_4,5_ edges both experimentally and theoretically. Hence, the transmission XMCD experiments of the samples in thin-film form are required. Such experiments, however, are virtually impossible in the BL23SU beamline, because the vaporization of uranium materials is not permitted in the SPring-8 facility.

The sum-rule analysis uses only the integrated MCD intensities and ignores the information contained in the spectral shape. In Fig. 7 ▶, atomic calculations adopting the intermediate coupling scheme for the U 5*f* ^2^ ground state (van der Laan & Thole, 1996 ▶) are compared. Although the experimental and theoretical curves possess the same qualitative features, there are some discrepancies in the XMCD structure at the *N*_5_ edge. The apparent discrepancies are attributable to additional solid-state effects. In metallic uranium compounds, XMCD spectra at the *N*_5_ and *M*_5_ edges display a significant variation in shape (Antonov *et al.*, 2008 ▶). Further analysis is currently under way (Takeda *et al.*, 2012 ▶).

## 5. Summary

The new twin-helical undulator, with the capability of switching the photon helicity, installed in BL23SU at SPring-8 is now operational. This device has improved the XMCD sensitivity by an order of magnitude. The experiments performed on paramagnetic β-US_2_ demonstrate the usefulness and promise of this technique. XMCD spectroscopy using lock-in detection methods is planned to allow more detailed studies. The new light source combined with on-going upgrades will elevate this beamline to an advanced instrumentation level and therefore greatly expand the scientific scope.
